# Long-Term Clearance and Biodistribution of Magnetic Nanoparticles Assessed by AC Biosusceptometry

**DOI:** 10.3390/ma15062121

**Published:** 2022-03-14

**Authors:** Guilherme A. Soares, João V. C. Faria, Leonardo A. Pinto, Andre G. Prospero, Gabriele M. Pereira, Erick G. Stoppa, Lais P. Buranello, Andris F. Bakuzis, Oswaldo Baffa, José R. A. Miranda

**Affiliations:** 1 Department of Biophysics and Pharmacology, Institute of Biosciences, São Paulo State University—UNESP, Botucatu 18618-689, SP, Brazil; joao.faria@unesp.br (J.V.C.F.); leonardo.antonio@unesp.br (L.A.P.); andre.prospero@unesp.br (A.G.P.); gabriele.martinsp@gmail.com (G.M.P.); e.stoppa@unesp.br (E.G.S.); lais.buranello@unesp.br (L.P.B.); jose.r.miranda@unesp.br (J.R.A.M.); 2 Institute of Physics, Federal University of Goiás, Goiânia 74690-900, GO, Brazil; abakuzis@gmail.com; 3 Faculty of Philosophy, Sciences and Letters at Ribeirão Preto, University of São Paulo, Ribeirão Preto 14040-900, SP, Brazil; baffa@usp.br

**Keywords:** magnetic nanoparticles, alternate current biosusceptometry, clearance, biodistribution, long-time analysis

## Abstract

Once administered in an organism, the physiological parameters of magnetic nanoparticles (MNPs) must be addressed, as well as their possible interactions and retention and elimination profiles. Alternating current biosusceptometry (ACB) is a biomagnetic detection system used to detect and quantify MNPs. The aims of this study were to evaluate the biodistribution and clearance of MNPs profiles through long-time in vivo analysis and determine the elimination time carried out by the association between the ACB system and MnFe_2_O_4_ nanoparticles. The liver, lung, spleen, kidneys, and heart and a blood sample were collected for biodistribution analysis and, for elimination analysis, and over 60 days. During the period analyzed, the animal’s feces were also collectedd. It was possible to notice a higher uptake by the liver and the spleen due to their characteristics of retention and uptake. In 60 days, we observed an absence of MNPs in the spleen and a significant decay in the liver. We also determined the MNPs’ half-life through the liver and the spleen elimination. The data indicated a concentration decay profile over the 60 days, which suggests that, in addition to elimination via feces, there is an endogenous mechanism of metabolization or possible agglomeration of MNPs, resulting in loss of ACB signal intensity.

## 1. Introduction

Over the past few years, there has been increased use of magnetic nanoparticles (MNPs) in a range of biomedical applications, such as drug delivery, in vivo cell tracking, diagnostics, magnetic resonance imaging (MRI), and thermal ablation therapy [1,2,3,4]. Due to their advantages in several biomedical uses and the possibility to manipulate them according to their use, which improves the interactions with the biological systems, many studies have been developed to reach all MNP’s benefits [5,6]. Besides understanding the MNPs composition and the surface functionality, it is necessary to comprehend the characterization of the MNPs in a biological system, which is essential to truly address the implications in future human medical applications. In addition, the real feasibility of these applications depends directly on the biodistribution and toxicity profiles [7].

The great challenge of nanomedicine is to offer multifunctional nanosystems biocompatible and non-toxic with biological targets [8,9]. Nowadays, the development, use, and study of the interactions of MNPs with biological systems have significantly increased, in contrast to the number of studies towards biodistribution, toxicity, and clearance studies. This divergence may be mainly attributed to the variety of MNPs used and to the methodologies applied.

Different routes may be used to infuse MNPs in the biological system, with the intravenous administration (IV) and intraperitoneal injection acting as the main routes [10]. IV injection remains the standard method to inject MNPs due to the instantaneous response provided and the possibility of obtaining much pharmacokinetic information. MNPs can be intravenously infused to be guided to a specific site such as a tumor, increasing treatment efficiency. Additionally, the IV route is useful for improving drug delivery efficacy, reducing the possible cytotoxicity of nanoparticles [11].

The size of the MNPs is a crucial factor for the biodistribution process. Consistent reports in the literature have demonstrated that MNPs larger than 100 nm in hydrodynamic diameter are primarily taken up by organs such as the liver, spleen, and lungs [12]. The main factor which contributes to this specific uptake is the mononuclear phagocytic system (MPS), also known classically as the reticuloendothelial system (RES), a complex network of cells specialized in the removal of xenobiotic materials from the bloodstream, broadly localized in these organs [13,14]. On the other hand, small MNPs (<10 nm) are virtually eliminated through renal clearance [15]. Moreover, the biodistribution is directly dependent on other physicochemical properties MNPs, including surface charge and coating [16,17].

Concerning the biodistribution of MNPs by these organs, well-established specialized tissue-resident macrophages are the main cells responsible for the uptake of nanoparticles. In general, MNPs preferentially accumulate in the liver and spleen, which are responsible for the sequestration of more than 95% of the nanoparticles due to the phagocytosis performed by the Kupffer cells the macrophages of the splenic marginal zone, respectively [18,19,20]. The liver is a highly perfused organ and extremely important in the uptake of both endogenous and exogenous substances due to its high blood flow, presenting liver sinusoidal endothelial cells (LSEC), which are highly fenestrated. The liver still has Kupffer cells and the resident localized macrophages, responsible for the uptake and elimination of many materials from the bloodstream [21]. Several studies have reported the intensive MNPs uptake carried by the hepatic structures, inclusive of the MNPs used in this work [22,23,24,25]. The spleen has a very interesting microanatomy, which can act as an efficient sieve to filter any exogenous material. The spleen is highly permeable vasculature with endothelial fenestrations. Moreover, the splenic vasculature is arranged as a way to facilitate the contact of MNPs and macrophages. The splenic arteries enter the organ and are finished off in highly porous capillaries, making the blood reach the marginal and red pulp zones [26]. These zones are the central splenic region for the MNPs uptake due to macrophages that phagocytize the MNPs. Studies reported the phagocytize process in the splenic zones through histological assessments or different biodistribution assessments methods [27]. Immediately after the injection, the MNPs are subjected to the opsonization process, characterized by the adsorption of plasma proteins on MNPs surface, allowing them to be easily recognized by the macrophages. As a result, this array of protein around the MNPs surface, often known as protein corona, increases the hydrodynamic size of MNPs; they are significantly removed from the bloodstream [28,29]. Physicochemical characteristics of MNPs may strongly influence the composition and architecture of the protein corona. Surface modifications of the MNPs shell are commonly carried out to modify their performance with biological targets. Coating the MNP with organic or inorganic molecules is one of the strategies widely employed to avoid the interaction with biological compounds [30]. Recently, Prospero and coworkers related that the protein corona composition is strongly dependent on MNPs characteristics, mainly including the size, the coating, and the surface charge. Indeed, this arrangement of protein makes the MNPs recognizable as a new complex biological structure that determines their biodistribution and clearance [31].

To achieve the real translational MNPs potential (theranostic), once they are administered in an organism, they must be detected in vivo and real-time to assess physiological parameters [32]. Additionally, it is necessary to evaluate their possible interactions and retention and elimination profiles. Nowadays, concerns about toxicity, safety, biodistribution, and clearance have emerged due to several MNPs applications [8,33].

Although several MNP biodistribution studies have recently been published, the process of the uptake and consequent metabolization and degradation of MNPs by the MPS remains unknown by nanomedicine [34]. Some literature works reported that the long-term accumulation may be beneficial for imaging and therapeutic applications [35], acting as a T2 contrast enhancement agent in MRI, either as a tracer or marker of new imaging modalities such as magnetic particle imaging and ACB imaging [22]. However, extensive pre-clinical trials must be addressed for real and future clinical applications [32], once the long-term effects of the MNPs aggregation deposited in the liver and spleen are still unknown [13]. Additionally, it is considered that MNPs retention has side effects for periods up to 11 months in the organs [36,37].

Over the years, there have been 51 nano-based products available for the therapies and diagnostic approved by the Food and Drug Administration (FDA) or European Medicine Agency (EMA) [38]. Nine MNPs are currently used as imaging agents, iron deficiency in chronic kidney disease (CKD), and magnetic hyperthermia regarding the inorganic and metallic nanoparticles [38,39]. Initially, based on the clinical success tests, FDA approved several MNPs to be used as MRI contrast agents, such as Feridex^®^ (Bayer Healthcare), Resovist^®^ (Bayer Healthcare), Combidex^®^ (AMAG Pharma), Sinerem^®^ (Guerbet), Clariscan^®^ (Nycomed), and VSOP C184 (Ferropharm). However, all the formulations have been discontinued from the market by the FDA due to efficacy or safety concerns [38,39,40,41].

The toxicity and the biodistribution analysis have become an issue of concern and require extensive investigation [42]. Currently, the approval of MNP as any nanomedicines and drugs is regulated by FDA. The completed process involves efficacy, safety, and toxicity studies. Nevertheless, the FDA regimentation and approval process, as for any other regulated drug, a complete knowledge about the mechanisms of the interactions MNP with the biological system is not required [43].

Over the years, different imaging, spectroscopy, and magnetometry techniques have been used to detect and quantify the biodistribution of MNPs in animals. In addition to techniques such as MRI and MPI [41,44,45,46]. Alternating current biosusceptometry (ACB) is a biomagnetic detection system used to detect and quantify MNPs, recently employed in several biomedical applications [22,23,24,31,47].

Therefore, in this work, the study aimed to evaluate the biodistribution and clearance of MNPs profiles through long time analysis and determine the elimination time carried out by the association between the ACB system and MnFe_2_O_4_ nanoparticles coated with citrate (Cit-MnFe_2_O_4_ MNPs).

## 2. Materials and Methods

### 2.1. ACB System

The ACB system is a magnetic detector and has been recently used in several studies involving MNPs. The ACB system theory is based on the mutual induction between two induction and pickup coils coaxially arranged in a first-order gradiometer. If a current oscillating sinusoidally is applied along with the indication coils, an alternate magnetic field is generated as $\;H=H_{a}\sin\left(\omega t\right)$, where $H_{a}$ is the field amplitude and ω is the angular frequency. Then, the differential induced voltage ($\Phi_{1}-\Phi_{2}$ from the primary and secondary pickup coils) is detected and expressed according to Faraday’s law:

(1) $$V_{d}=-NA\frac{d\Phi }{dt}$$

When a sample is positioned at the center of one of the pickup coils, the magnetic flux induced by a sample with magnetization M(H) in a pickup coil with is:

(2) $$\Phi =-\mu_{0}NAM\frac{d}{dt}\left[\left(M+H_{a}\right)-H_{a}\right]$$

The magnetic flux can be rewritten as an ideal balanced detection coil system:

(3) $$\Phi =-\mu_{0}NAM\frac{d}{dt}\left[\left(M+H_{a}\right)-H_{a}\right]$$

From Equation (3), the final voltage detected results:

(4) $$V_{d}=-\mu_{0}NAM\frac{dM\left(t\right)}{dt}$$

In this way, the instrumental arrangement turns the system into a magnetic flux transformer. The coil pair (excitation/detection) furthest from the sample acts as a reference, while the closest to the magnetic material acts as detection.

**Figure 1:**
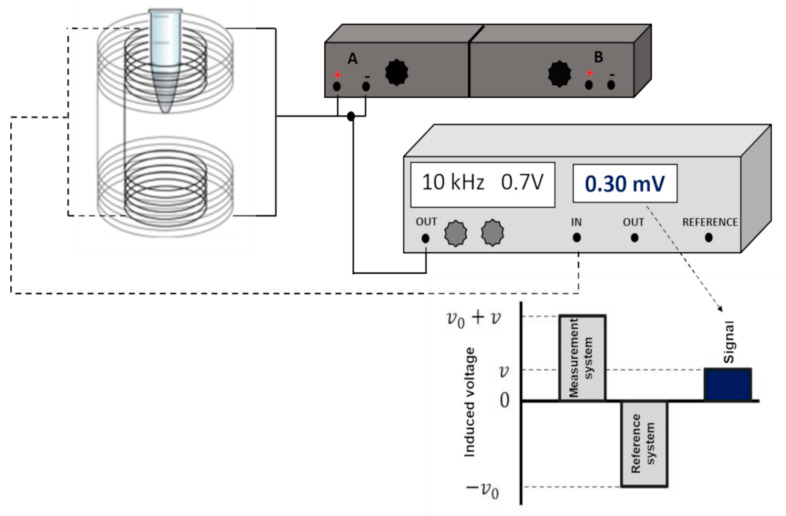
Schematic diagram of ACB setup used for MNP measurements. Through a phase-sensitive amplifier (lock-in—Stanford Research Systems SR830) (light grey), an electrical signal of 0.7 V at a frequency of 10 kHz is generated and is amplified by power amplifiers (−3 dB) (dark gray), in which the resulting current is applied to the excitation coils.

The MNP biodistribution and elimination signals quantification were carried out using an ACB setup already reported by [23,47]. As demonstrated previously, the setup presents high sensitivity and accuracy for ex vivo analysis. Figure 1 presents the schematic diagram of the ACB setup used for MNP measurements.

### 2.2. Magnetic Nanoparticles

We employed citrate-coated manganese ferrite (Ci-MnFe_2_O_4_), nanoparticles synthesized by a co-precipitation method previously characterized and described [31,47]. Ci-MnFe_2_O_4_ at a 23 mg/mL concentration presented a superparamagnetic behavior and a magnetization saturation of 264 kA/m. The MNPs characterization was performed by dynamic light scattering (DLS) Zetasizer NanoS (Malvern Instruments, Malvern, UK). and Transmission electron microscopy (TEM) (Jeol, Tokyo, Japan). All MNPs characterization can be found in the Supplementary material. Figure S1 shows the TEM images for the magnetic nanoparticles, and Figure S2 shows the particles size distribution. Figure S3 presents the hydrodynamic distribution for the magnetic nanoparticles, Figure S4 presents the magnetization curve of the manganese-ferrite nanoparticles, and Figure S5 shows the X-ray diffraction pattern of the citrate-coated manganese-ferrite nanoparticles.

### 2.3. Animal Experiments

All animal experiments were previously approved and performed following the Committee on Ethics in Animal Use, under the protocol (CEUA)–IBB 1135.

Fifty male rats weighing 250–300 g (Rattus norvegicus albinus, Wistar; acquired from the Anilab, Paulinia, SP, Brazil) were subjected to ten groups that were established by the animal euthanasia time: 1 h, 4 h, 12 h, 24 h, 48 h, 5 days, 10 days, 15 days, 30 days, and 60 days.

All the animals were subjected to the same experimental protocol. The animals were anesthetized with isofluorane (5% for induction and 2% for maintenance) and underwent cannulation surgery of the left femoral vein for intravenous administration of MNPs. The animals received a single injection of 0.3 mL of MNPs (total of 6.9 mg of MNPs) at an administration rate of 0.03 mL/s and were euthanized by decapitation, referring to the time point.

To assess the biodistribution pattern as a function of time, the liver, lung, spleen, kidneys, and heart were collected. In addition, a sample of blood and feces was also collected. After the experimental procedure, all the collected samples were submitted to a Labconco FreeZone 2.5 benchtop freeze dryer (Labconco, Kansas City, MO, USA and stored in a volume-controlled flask.

### 2.4. Ex Vivo Biodistribution and Pharmacokinetic Assessment

The feces of the animals were collected every 24 h and were then subjected to the same freeze-drying process for further analysis to assess the MNPs elimination profile.

To provide an ex vivo quantitative evaluation about the MNP distribution previously in vivo administered, we also built a calibration curve for the two MNPs used here to compare the ACB signal obtained with ACB response to a known concentration of samples, owning a well-established mass of MNPs. To understand how MNP features and physiology can influence the liver MNP accumulation pattern, it was proposed to investigate the biodistribution data obtained from the ACB analysis through a pharmacokinetic model. Therefore, the MNP half-life in the bloodstream ($T_{1/2}$) can be modeled according to Equation (5):

(5) $$Y_{\left(t\right)}=Y_{0}+A_{1}e^{-t/\tau 1}+e^{-t/\tau 2}$$

Equation (5) assumes that $Y_{0}\;$ corresponds to the ACB signal immediately before the injection, and *τ*1 and *τ*2 refer to the two average elimination exponential coefficients. At the same time, the parameters $A_{1}$ and $A_{2}$ (uptake indices), when summed, represent the total MNP accumulation at each instant. Regarding the spleen MNP clearance, its elimination was modeled using the following Equation (6):

(6) $$Y_{\left(t\right)}=Y_{0}+A_{1}e^{-t/\tau 1}$$

Statistical calculations and half-life quantifications were performed using OriginLab 8.5 (Version 2016, OriginLab Corporation, Northampton, MA, USA).

## 3. Results

### 3.1. MNPs Characterization

**Figure 2:**
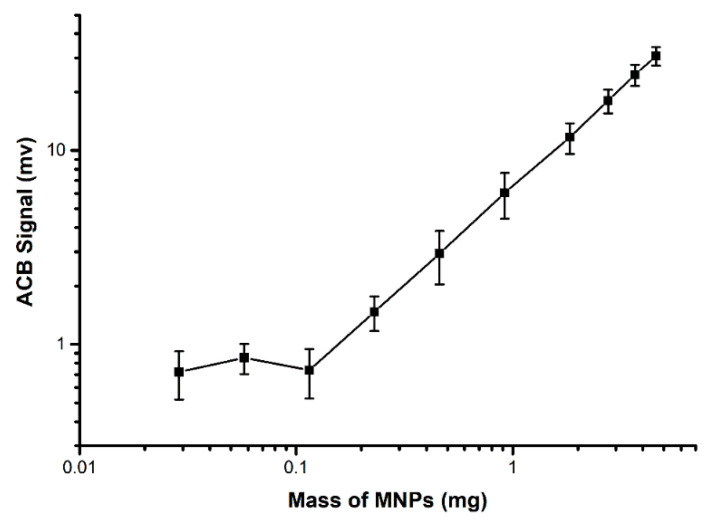
Calibration curve of citrate-coated MNPs in linear scale with linear fits, where an R^2^ = 0.99 was obtained for citrate-coated MNPs.

Two methodologies were employed for the MNPs characterization. The hydrodynamic diameter was determined, followed by an analysis of ACB signal response to different concentrations of MNPs. Through the DLS analysis, MNPs hydrodynamic diameter and zeta potential were 40 ± 5.6 nm and −27.8 mV, respectively. The MNPs had a polydispersity index of 0.175 ± 0.092. Figure 2 shows the calibration curve and the linear response of the ACB system for citrate-coated MNPs.

### 3.2. MNPs Biodistribution and Elimination

**Figure 3:**
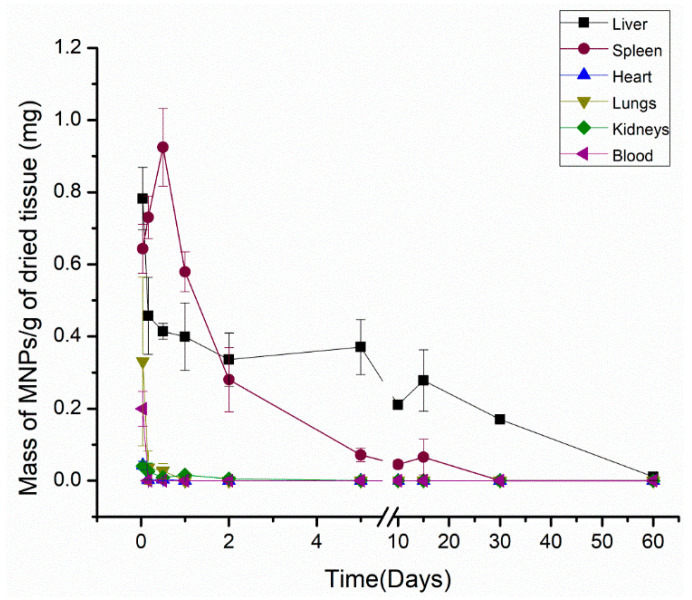
Biodistribution results for all organs of interest of the citrate-coated MNPs over the period evaluated.

Figure 3 shows the biodistribution of MNP Cit-MnFe_2_O_4_ in each organ, quantified from one hour until 60 days after the in vivo administration.

As depicted in Figure 3, besides the predominant accumulation in the liver and spleen, MNPs were in all the organs from 1 h and until 12 h after the injection. In the liver, MPNs were detected along the entire measured period (60 days), presenting a maximum amount of MNP one hour after administration (5.4 mg of MNPs). At measurement times determined around 60 days, the amount of MNP reached low levels, around zero. MNPs were significantly detected within 15 days after in vivo administration regarding the spleen, which presented the highest accumulation MNPs around 12 h (0.092 mg). Similar to the liver, the spleen MNPs signal tends to decrease with time, which can detect a very low mass of MNPs.

Despite the low ACB signal intensity, MNPs could be seen in the kidneys until 48 h. Both the heart and the lungs accumulated MNP for only 12 h, showing a maximum ACB signal of the MNP injection in one hour after the injection, which is the only time that it was possible to detect MNP circulating in the blood.

The data obtained through the quantification of the ABC system indicated that the elimination kinetics from the infusion of MNPs occur according to an exponential behavior. As the liver and spleen are considered the two main organs responsible for MNPs uptake from the bloodstream, the pharmacokinetics of the MNP pattern for both organs were compared, employing a bi-exponential model to determine the MNPs circulation half-life.

**Figure 4:**
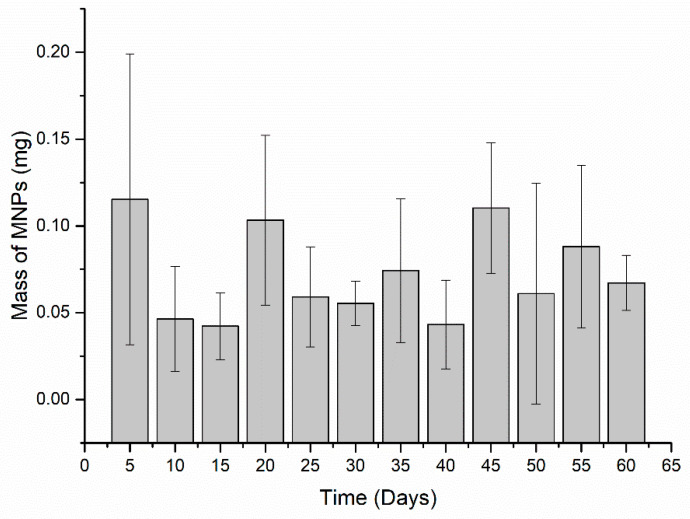
Elimination of MNPs via feces every five days. For statistical analysis, the Mann–Whitney U test was used. It was found no significant difference between the days (*p* < 0.05).

In a rat model, the Cit-MnFe_2_O_4_ exhibited biexponential liver concentration decay, with a half-life of 70 min for the initial phase, which is faster and responsible for the distribution and clearance for most of the injected dose. In contrast, the second phase is slower and presents a half-life of 30 days. Regarding the spleen half-life, a single-phase $T_{1/2\;}$ of 1.75 days was found. To quantify the amount of MNPs in the collected feces, we established a protocol to quantify the samples in five days (Figure 4)

**Figure 5:**
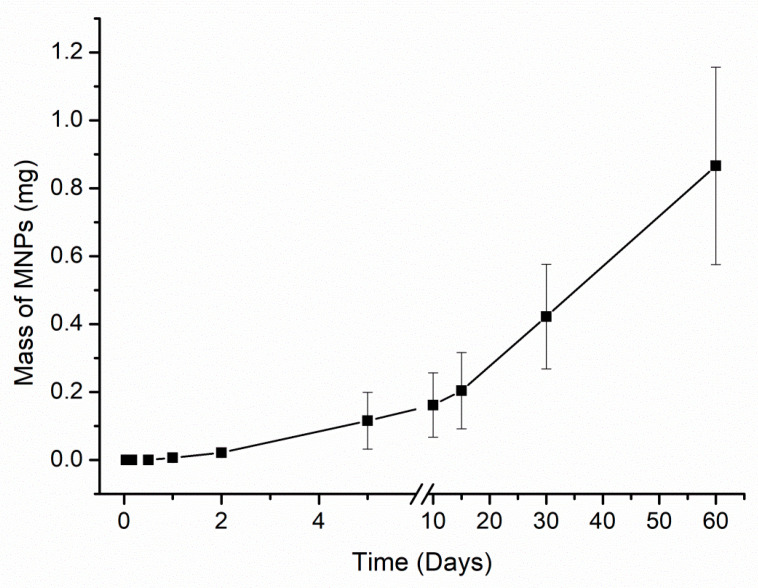
Cumulative elimination profile of Cit-MnFe_2_O_4_ MNPs via feces over the period evaluated.

The highest elimination day occurred five days after administration, presenting 0.115 ± 0.08 mg MNPs eliminated. Although this initial period of five days showed the highest elimination values, no elimination pattern was noticed, considering that the values do not differ much from the other values found. Moreover, the elimination profile presents an approximate amount MNPs within 0.05 and 0.1 mg every five days. Additionally, the accumulated profile of MNPs elimination was assessed. Figure 5 shows the total of MNPs eliminated over the entire period.

Figure 5 indicates that the MNPs elimination precisely starts from two days after administration. It is worth pointing out that all animal feces were collected before euthanasia, and all feces were analyzed individually. The total clearance was 0.87 ± 0.29 mg MNPs.

## 4. Discussion

This study employed the ACB system to assess the biodistribution and elimination pattern via feces of Cit-MnFe_2_O_4_ MNPs over long periods after intravenous administration in rats. The soft-ferrite based MNPs were used due to their excellent low-field magnetic response. Moreover, the MNPs system has high magnetization saturation which, in association with ACB system configuration of magnetic field of 2 mT and frequency of 10 kHz, presents high magnetic susceptibility and consequently a good detection. Additionally, the Cit-MnFe_2_O_4_ MNPs present suitable properties towards to magnetic hyperthermia [48,49].

Under the perspective to ensure safety future in vivo applications, it is still mandatory to assess the MNPs’ time-dependent biodistribution and clearance [50,51]. In this way, our results for Ci-MnFe_2_O_4_ MNPs distribution and clearance made it possible to observe a predominant retention profile in the liver and spleen. The highest uptake of both organs is mainly due to morphophysiological characteristics combined with specialized structures for filtration and retention in these organs.

As can be seen 15 days after the administration, the concentration of MNPs over time in the spleen showed a slight increase in its concentration, which may be correlated with the same rise in liver concentration, indicating that the two organs can act similarly, most likely due to their characteristics.

We also quantified the two half-lives of the liver elimination and the spleen half-life elimination through the pharmacokinetic assessment. Regarding the hepatic clearance, the elimination time was evaluated by a two-phase $\;T_{1/2}$. The first half-life found can be assigned to the primary MNPs filtration performed by the liver, which captured a high MNP amount. The following half-life reflects the bi-exponential exchange that the liver and bloodstream carry out where part of the MNP returns to the bloodstream. On the other hand, the spleen presented only a phase $\;T_{1/2}$, characterized by an intense decay. Despite being a highly perfused organ and its high number of macrophages, the spleen did not show the same clearance behavior as the liver; we noticed that around the second post-MNP day injection, the spleen eliminated most particles at once.

MNPs were significantly detected in the blood in the first hour after administration. However, we found no MNPs in the blood four hours after injection, which may be correlated with the first depuration of the MPS system, removing most of the circulating particles. Quini et al., 2017 [25] showed no signal in the blood four hours after MNPs infusion. This behavior suggests that all MNPs have already been captured by organs or tissue at this time. The low intensity of MNPs present in the heart, and its subsequent decay four hours after administration, can be related to the presence of particles, in the same time interval, in the blood. The absence of MNPs in the heart may be related to particles in the bloodstream and the heart’s characteristics, as it does not present retention characteristics. Although the heart is an excessively irrigated organ, the corona effect in the blood may be responsible for avoiding the internalization of MNPs by cardiac cells.

Despite being highly perfused and with their own resident macrophages (myeloid cells residing in renal tissue and alveolar macrophages in the lung tissue) [52,53], the kidneys and the lungs presented a considerably lower signal intensity than the spleen and liver. Studies showed that, despite the morphological characteristics of both organs, there is a dimensional and surface charge dependence for MNPs uptake, which did not contribute to MNP uptake by the liver and lungs. It was also hypothesized that a group of proteins could have bound to the MNP surface, improving the corona protein, which increased their size and facilitated the recognition and the subsequent molecular interactions with the hepatic and splenic structure.

In our finding, it was noticed a decrease in the ACB signal over the time analyzed, which can be directly associated with the MNP state several days after the administration. From this, it is noteworthy that the ACB system is strongly affected by the MNP condition. Therefore, the signal decrease would be explained by inhibiting the Brownian relaxation due to the arrangement of proteins around the MNP surface, resulting in altered magnetic susceptibility. Concomitantly, it is noticeable that partial metabolization after a few days of the MNP infusion also influences the ACB signal and contributes to the signal decrease once the ACB system can detect the MNP in its molecular form, so that no metabolites or ions from MNPs could be seen. The MNP partial metabolization process can be verified through Figure 5, in which was detected around 14% of the injected dose in the final measurement period. It was considered that most MNPs were metabolized or degraded by biological mechanisms that induced changes in the magnetic properties of nanoparticles.

Long-term biodistribution studies illustrate the difficulty of eliminating nanostructured materials when administered to the body, reinforcing the importance of this study modality for future clinical applications of MNPs. Table 1 summarizes studies dedicated to monitoring the MNP’s long-term biodistribution and the respective animal models and methodologies used.

As depicted in Table 1, several approaches have been used to assess the accumulation of different MNPs. Regardless of the type of MNPs, these nanomaterials were detected mainly in the livers of different species over days and months.

Mejías et al., 2013 [54] detected iron oxide MNPs within a period of 30 min to 90 days. However, this study did not establish the total elimination period as, at 90 days, significant amounts of MNPs were still present in the organs. Similar to our findings, Ruiz et al., 2015 [55] analyzed the biodistribution of magnetite nanoparticles up to 30 days post-administration. The study could observe a significant accumulation of MNPs in organs such as the liver, spleen, and lung within 30 days. In another study, Yang and collaborators also found a high concentration of iron seven days after administration of iron oxide nanoparticles in organs such as the liver, spleen, and kidneys [56]. Nonetheless, analyses based on Fe concentrations can induce a series of variations, mainly due to the endogenous iron itself and that MNP may have been metabolized, presenting only an ionic form.

On the other hand, Tate et al., 2011 [32] carried out a long-term assessment in which the authors reported the presence of iron oxide nanoparticles in the liver 580 days after the injection. Other studies also examined the distribution patterns of the non-magnetic nanoparticles and found significant nanoparticles amounts more than one month in organs responsible for the elimination process.

In this way, we must take into consideration that the MNPs are not eliminated simply as common drugs, as they can remain in the body for months according to our evaluation and literature data. MNPs’ time course assessments become crucial to provide the MNPs as a promising material for imaging and therapy applications in medicine.

## 5. Conclusions

Cit-MnFe_2_O_4_ MNPs were primarily accumulated in the liver and spleen due to these organs’ morphological and physiological characteristics and the intrinsic MNPs characteristics.

The data obtained made it possible to observe a concentration decay profile over the 60 days, which suggests that, in addition to elimination via feces, there is an endogenous mechanism of metabolization or possible agglomeration of MNPs, resulting in loss of ACB signal intensity. Furthermore, the clearance profile for MNPs was assessed over the measured period.

The ACB system offers a low-cost, portable, and versatile alternative for evaluating the biodistribution and elimination of MNPs, even at low concentrations. Regarding elimination, the data presented in this work suggest that the MNPs used demonstrated a constant elimination rate that starts from 48 h via feces, which can assist in providing strategies to target drug delivery to specific cell types and consolidate them as agents for magnetic hyperthermia.

Even with the clearance and elimination studies, future studies are required to deeply elucidate the toxicity and the specific elimination mechanisms, aggregation, and metabolization of MNPs. Additionally, the ACB system is a feasible methodology to be employed with different modalities to understand the mechanism of metabolization and clearance of MNPs fully.

## Figures and Tables

**Table 1:** Studies employed on assessment of the MNPs long-term biodistribution.

MNPs	Species	Dose	Time Post-Injection Assessed	Method/Technique	Ref.
DMSA-coated magnetite	C57BL/6 mice	15 mg Fe kg^−1^	90 days	Quantum Design MPMS-XL SQUID magnetometer/ICP-AES	[54]
DMSA/PEG Magnetite DMSA	Wistar rats	2.5 mg Fe/kg B.W	30 days	ICP-OES	[55]
Carboxyl coated Iron Oxide	KunMing mice	20 mg kg^−1^	7 days	Atomic absorption spectroscopy	[56]
Dextran-coated iron oxide nanoparticles	C3H mice	2 mg Fe/mouse	580 days	Histological analysis/ICP-MS	[32]
Citrate coated MnFe_2_O_4_	Wistar rats	Multiple injections of 6.9 mg/rat	24 h	AC Biosusceptometry/Electron spin resonance	[25]
Dextran-coated magnetite	Swiss mice	100 μL (1 × 10^17^ particle/mL)	6 months	Magnetic resonance	[52]
Curcumin capped iron oxide nanoparticles	Balb/c mic	5 mg kg^−1^	3 weeks	Atomic absorption spectroscopy.	[8]
Maghemite coated by hydrophilic derivatives of glucose	mice (C57-B6 mice)	1000 μmol of iron kg^−1^ and 50 μmol of iron kg^−1^	3 months	EPR and SQUID	[53]
Maghemite (γ-Fe2O3)	Swiss mice	2.4 mg iron	28 days	ICP OES and histological methods.	[57]
γ-Fe2O3 s-SPION	Nude mice (BALB/c-Foxn1nu/Arc)	90 mg Fe kg^−1^	7 days	Atomic absorption spectroscopy (AAS) and Prussian blue	[33]
Dextran-Iron oxide nanoparticles	Wistar rats	10 mg kg^−1^	28 days	ICP-AES	[58]
Iron oxide nanoparticles	BALB/c mice	5 mg/mL	24 h	ICP-MS	[7]
Dextran-coated magnetite	Swiss mice	100 μL/mice	6 months	Magnetic resonance	[59]
Iron oxide NPs (Fe_2_O_3_)	Wistar rats	7.5 mg/kg, 15 mg/kg and 30 mg/kg	28 days	Atomic absorption spectroscopy (AAS)	[60]
Ferucarbotran	Fisher 344 female rats	5 mg Fe/kg	70 days	MPI	[61]

## Data Availability

Almost all data are presented within the manuscript (Figures and Tables). The raw data presented in this study are available on request from the corresponding author.

## Supplementary Materials

The following are available online at https://www.mdpi.com/article/10.3390/ma15062121/s1, Figure S1: TEM images for the magnetic nanoparticles. Scale: (Upper) 100 and (Lower) 10 nm, Figure S2: Particles size distribution, Figure S3: Hydrodynamic distribution for the magnetic nanoparticles, Figure S4: Magnetization curve of the manganese-ferrite nanoparticles, Figure S5: X-ray diffraction pattern of the citrate-coated manganese-ferrite nanoparticles. References [62,63] are sited in the supplementary materials.
