# Supplementary material for: Long-Term Clearance and Biodistribution of Magnetic Nanoparticles Assessed by AC Biosusceptometry
Source: Materials (Basel). 2022 Mar 14;15(6):2121. doi: 10.3390/ma15062121 (PMC8948936; doi:10.3390/ma15062121)
Supplement: Supplementary file 1 [file materials-15-02121-s001.zip › materials-1495030-supplementary.pdf]

materials

MDPI

Article

Long-Term Clearance and Biodistribution of Magnetic NanoParticles Assessed by AC Biosusceptometry

Guilherme A. Soares [1,*], João V.C.Faria [1], Leonardo A. Pinto [1], Andre G. Prospero [1],Gabriele M. Pereira [1], Erick G. Stoppa [1], Lais P. Buranello [1], Andris F. Bakuzis [2] and José Ricardo de Arruda Miranda [1]

1 Department of Biophysics and Pharmacology, Institute of Biosciences, São Paulo State University—UNESP, Botucatu, São Paulo 18618-689, Brazil; joao.faria (J.V.C.F.); leonardo.antonio@unesp.br (L.A.P.); gabriele.martinsp@gmail.com (G.M.P.); andre.prospero@unesp.br (A.G.P.); e.stoppa@unesp.br (E.G.S.); lais.buranello@unesp.br (L.P.B.); jose.r.miranda@unesp.br (J.R.A.M.)

2 Institute of Physics, Federal University of Goiás, Goiânia 74690-900, Brazil: abakuzis@gmail.com (A.F.B)

* Correspondence: guilherme.soares@unesp.br

Citation: Soares, G.A.; Faria, J.V.C.; Pinto, L.A.; Prospero, A.G.; Pereira, G.M.; Stoppa, E.G.; Buranello, L.P.; Bakuzis, A.F.; Baffa, O.; Miranda, J.R.d.A. Long-Term Clearance and Biodistribution of Magnetic Nanoparticles Assessed by AC Biosusceptometry. *Materials* **2021**, *15*, 2121. https://doi.org/10.3390/ma15062121

Academic Editor(s): Tatyana I. Shabatina and Olga I. Vernaya

Received: date
Accepted: date
Published: date

Publisher's Note: MDPI stays neutral with regard to jurisdictional claims in published maps and institutional affiliations.

Copyright: © 2021 by the authors. Submitted for possible open access publication under the terms and conditions of the Creative Commons Attribution (CC BY) license (http://creativecommons.org/licenses/by/4.0/).

The magnetic nanoparticles (MNPs) used were synthesized by a previously described coprecipitation procedure [1]. The MNPs consist of manganese ferrite-based nanoparticles coated with citrate. Regarding the toxicity, the MNP system has already been tested and presented safety for in vivo application under the concentration used in this work.[2] Through a JEOL model JEM-2100 transmission electron microscope (TEM) (Tokyo, Japan), operating at 200 kV (2.5 Å resolution), the MNPs' core diameter distribution was determined. Figure S1 presents the TEM images.

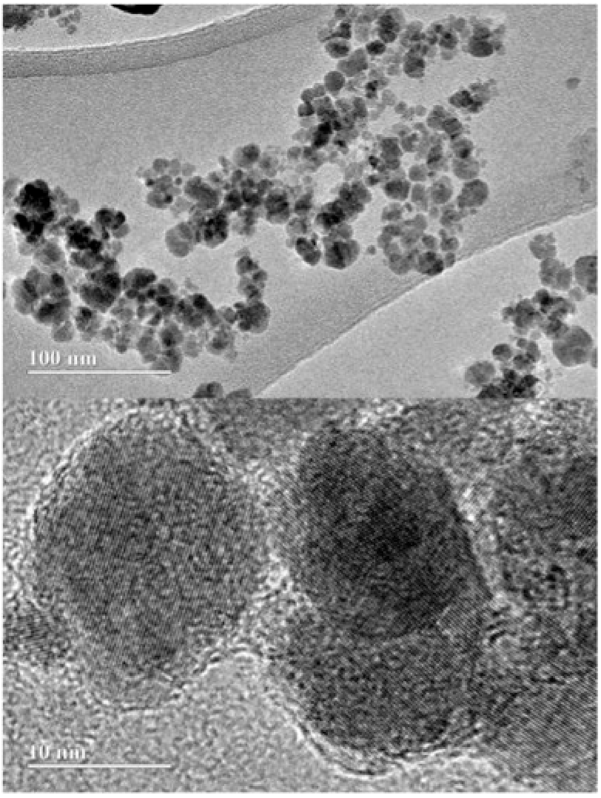

Figure S1. TEM images for the magnetic nanoparticles. Scale: (Upper)100 and (Lower)10 nm.

From the TEM images obtained and using the software ImageJ, it was assessed through a histogram generated (Figure S2).

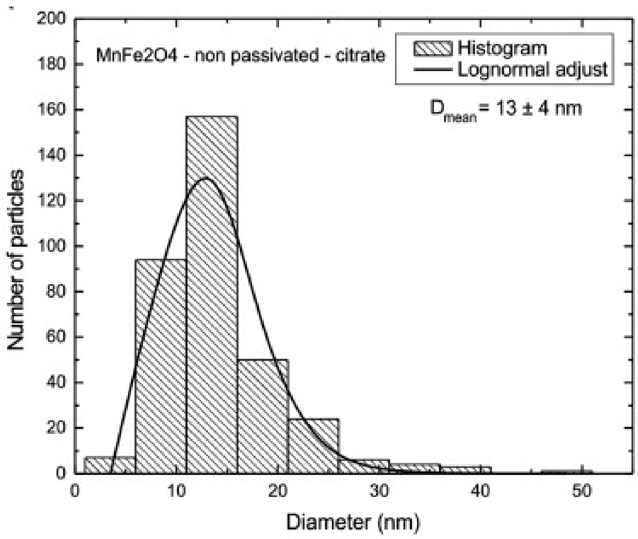

Figure S2. Particles size distribution.

The hydrodynamic radius (HD) and polydispersion index (PDI) were determined using a Zetasizer NanoS (Malvern Instruments, Malvern, UK), in which the values were 40 ± 5.6 nm and 0.75, respectively. Figure S3

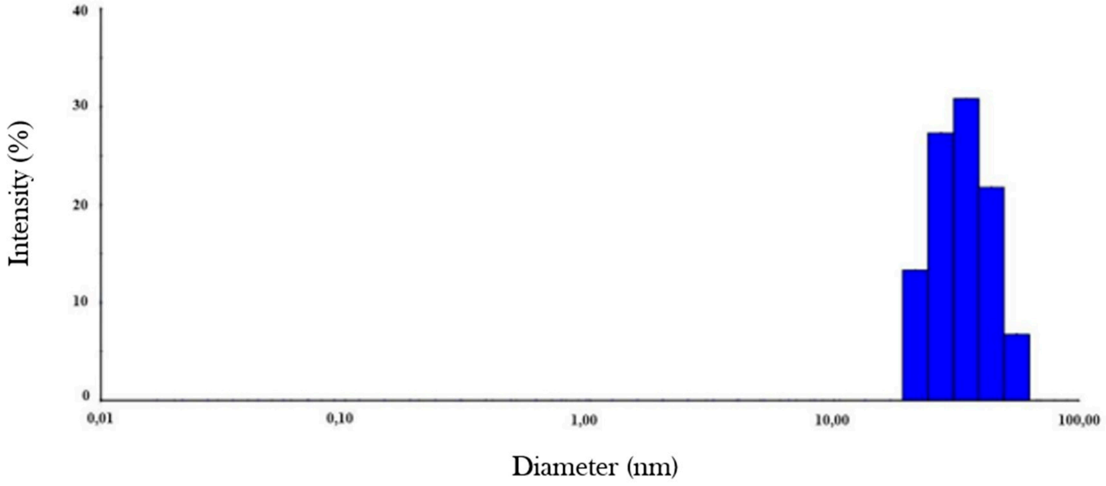

Figure S3. Hydrodynamic distribution for the magnetic nanoparticles.

The magnetization results were acquired through an ADE Vibrating Sample Magnetometer model EV9 (MicroSense, EastLowell, MA, USA). The saturation magnetization of the nanoparticles was found to be 52.8 emu/g (264 emu/cm^3), and it is possible to notice that the data indicates that the nanoparticles behave as a quasi-static superparamagnetic behavior (Figure S4).

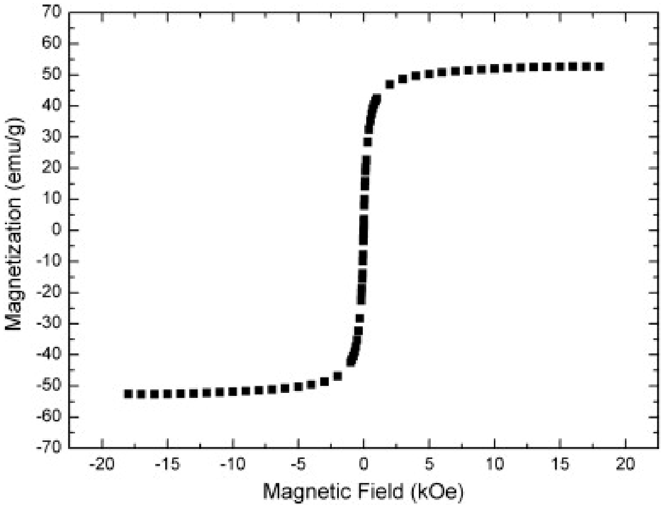

Figure S4. Magnetization curve of the manganese-ferrite nanoparticles.

The manganese ferrite-based nanoparticles coated with citrate composition were provided by Energy-dispersive x-ray spectroscopy (EDS) using an EDS detector coupled to the TEM system. MNP samples were fixed in a copper screen coated with carbon, and fourteen measurements were conducted using five MNPs samples. Fe and Mn content were 74.4 ± 2.6% and 25.6 ± 2.6%, respectively (Figure S5).

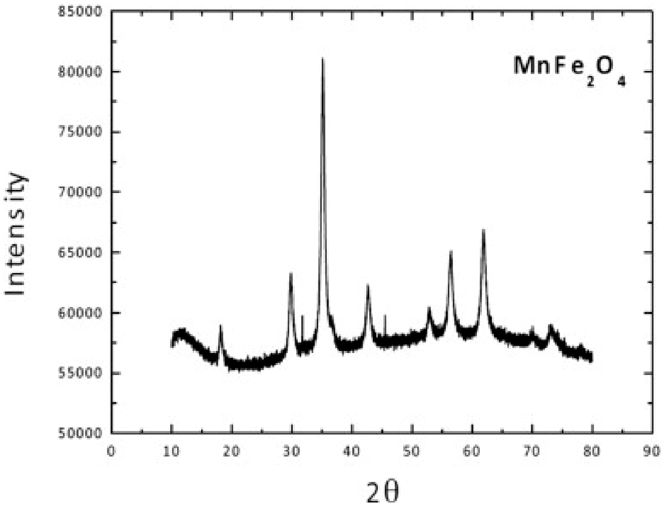

Figure S5. X-ray diffraction pattern of the citrate-coated manganese-ferrite nanoparticles.

References

Quini, C.C.; Matos, J.F.; Próspero, A.G.; Calabresi, M.F.F.; Zufelato, N.; Bakuzis, A.F.; Baffa, O.; Miranda, J.R.A. Renal perfusion evaluation by alternating current biosusceptometry of magnetic nanoparticles. *J. Magn. Magn. Mater.* **2015**, *380*, 2–6, doi:https://doi.org/10.1016/j.jmmm.2014.09.073.

Nunes, A.D.; Ramalho, L.S.; Souza, Á.P.; Mendes, E.P.; Colugnati, D.B.; Zufelato, N.; Sousa, M.H.; Bakuzis, A.F.; Castro, C.H. Manganese ferrite-based nanoparticles induce ex vivo, but not in vivo, cardiovascular effects. *Int. J. Nanomedicine.* **2014**, *9*, 3299–3312.
